# Erythrasma Mimicking Tinea Versicolor: The Diagnostic Utility of Ultraviolet-Induced Fluorescence Dermoscopy and Successful Combination Therapy

**DOI:** 10.7759/cureus.96970

**Published:** 2025-11-16

**Authors:** Balachandra S Ankad, Sourab Dhananjaya

**Affiliations:** 1 Dermatology, S Nijalingappa Medical College, Bagalkot, IND; 2 Dermatology, Subbaiah Institute of Medical Sciences and Research Centre, Shivamogga, IND

**Keywords:** corynebacterium minutissimum, erythrasma, tinea versicolor, ultraviolet-induced fluorescence dermoscopy, wood's lamp

## Abstract

Erythrasma is a chronic superficial bacterial infection caused by *Corynebacterium minutissimum*, often misdiagnosed as dermatophytosis or pityriasis versicolor due to overlapping morphology. A 55-year-old gentleman presented to our Dermatology Department with itchy skin lesions that had progressed slowly over three years. On examination, scaly, hyperpigmented patches were noted, localized to the axillae and groins. He had taken multiple courses of antifungals without relief. Potassium hydroxide (KOH) examination showed no spores or hyphae; polarized dermoscopy demonstrated brown areas with scaling, and ultraviolet-induced fluorescence (UVF) dermoscopy showed coral-red fluorescence consistent with erythrasma. He received topical Whitolyn (salicylic acid 3% plus benzoic acid 6%) mixed with 2% fusidic acid twice daily, along with oral doxycycline 100 mg once daily for 20 days, and demonstrated significant clinical improvement, with complete disappearance of coral-red fluorescence on repeat UVF dermoscopy. UVF dermoscopy, by enhancing porphyrin fluorescence produced by *C. minutissimum*, serves as a rapid, non-invasive diagnostic and follow-up tool. It allows clear differentiation of erythrasma from mimickers such as tinea versicolor or candidiasis, which lack such fluorescence, thereby aiding in both accurate diagnosis and assessment of therapeutic efficacy.

## Introduction

The superficial bacterial infection of the stratum corneum known as erythrasma results from *Corynebacterium minutissimum*, which exists as part of the normal skin flora but becomes pathogenic under conditions such as heat, moisture, and occlusion [1,2]. The condition appears most often in intertriginous areas, including the axillae, groin, under the breasts, and between the toes, and presents as well-defined reddish-brown scaly spots that may either cause itching or remain asymptomatic [1,3]. It is frequently mistaken for tinea versicolor, candidiasis, or intertrigo, which often leads to inappropriate antifungal treatment and prolonged disease [1,4].

Erythrasma occurs most often in tropical and subtropical regions, while people who have diabetes, obesity, and weakened immune systems tend to develop this condition more frequently [2,5]. The pathogenic bacteria create coproporphyrin III, which produces a distinct coral-red fluorescence when examined under Wood’s lamp, making it a traditional diagnostic marker [3]. The diagnostic process becomes complicated because Wood’s lamp evaluation reveals restricted tissue morphology and produces variable results based on environmental light conditions and additional infections [4,6]. Conventional potassium hydroxide (KOH) microscopy and Wood’s lamp examination may fail to discriminate erythrasma from superficial fungal infections when mixed colonization occurs.

Recent advances with better accuracy, such as ultraviolet-induced fluorescence (UVF) dermoscopy, which unites magnification with UV illumination to detect specific fluorescence patterns [6,7]. The UVF dermoscopy of erythrasma displays red fluorescence that forms polygonal shapes and structureless patterns, which results from porphyrin buildup, thus helping to distinguish it from fungal skin conditions like tinea versicolor [4,7].

Erythrasma usually gets better with topical or systemic antibacterial treatment, which includes macrolides, clindamycin, and fusidic acid, but clinicians often encounter challenges with chronic and recurrent cases because they frequently misdiagnose and under-treat these patients [5]. This case report describes a long-term erythrasma case, which doctors initially mistook for tinea versicolor before UVF dermoscopy confirmed the diagnosis, demonstrating how this diagnostic method helps clinicians differentiate bacterial from fungal infections for proper treatment [5].

## Case presentation

**Figure 1:**
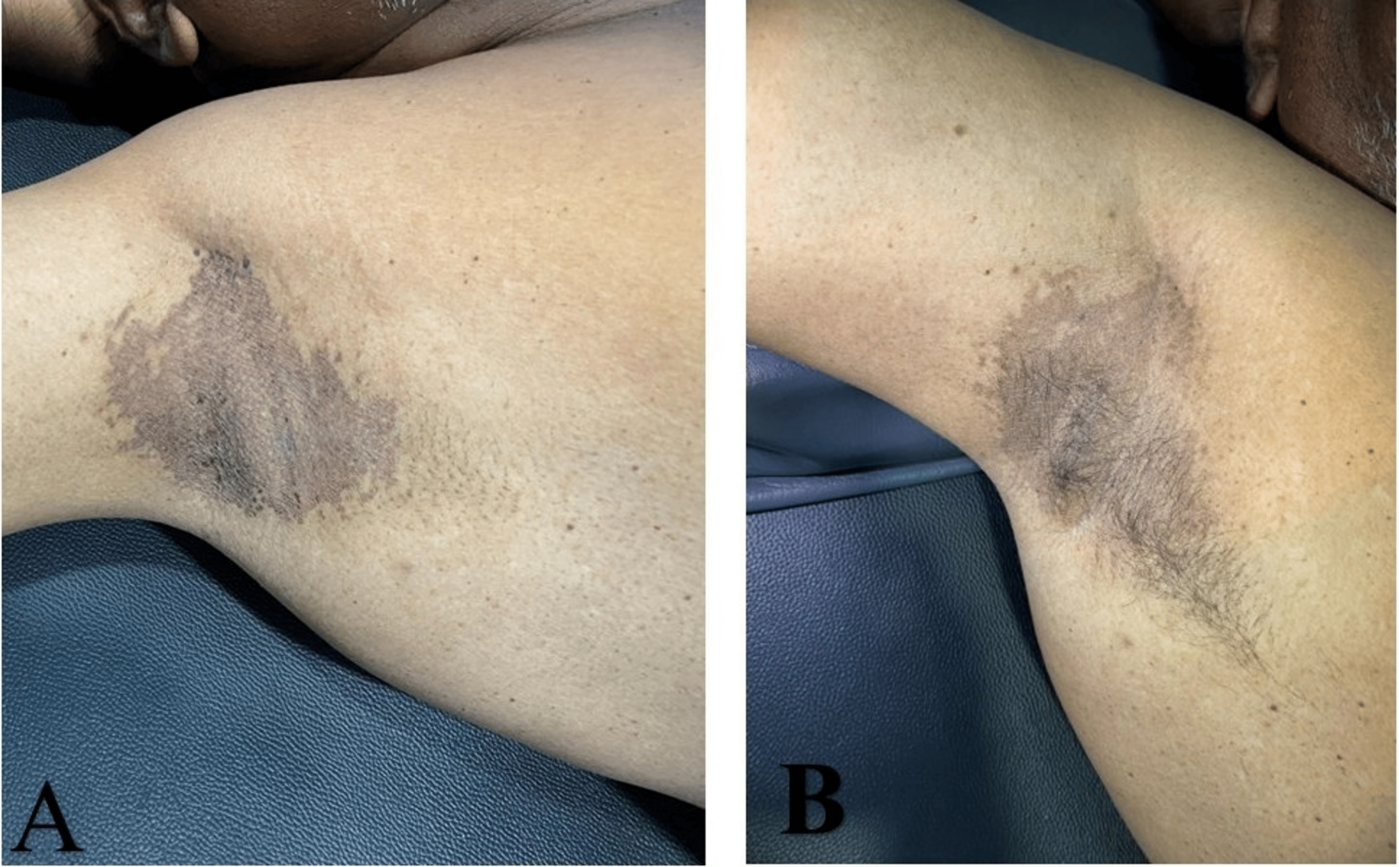
Clinical photographs of axilla A) Pre-treatment clinical photograph showing well-defined, hyperpigmented scaly patches in the axilla characteristic of erythrasma. B) Post-treatment clinical photograph demonstrating marked resolution of lesions following therapy.

**Figure 2:**
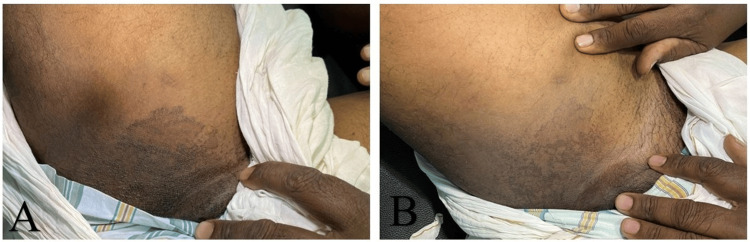
Clinical photographs of groin A) Pre-treatment clinical photograph of erythrasma involving the groin showing hyperpigmented scaly plaques. B) Post-treatment clinical photograph showing near-complete clearance of groin lesions after therapy.

A 55-year-old gentleman presented with mildly pruritic, hyperpigmented, scaling lesions over the axilla (Figure 1A) and thighs (Figure 2A) for the past three years. Multiple antifungal and topical treatments had been instituted several times previously for presumed tinea versicolor, without success. There was a slow extension of the lesions; however, he had no systemic symptoms. He was non-diabetic, non-obese, and maintained adequate hygiene.

**Figure 3:**
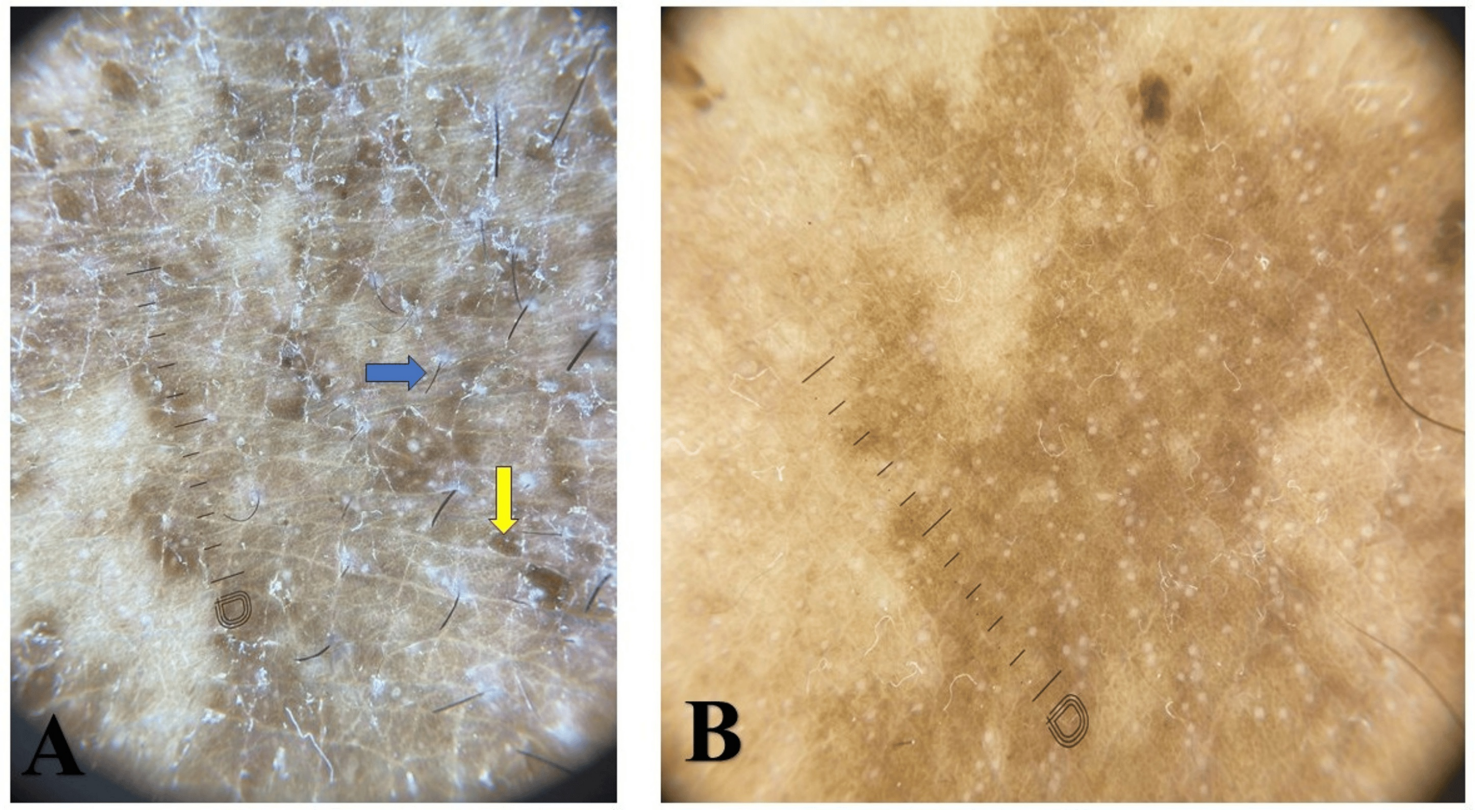
Polarized dermoscopy image A) Pre-treatment polarized dermoscopic image (DermLite DL5, with 10× magnification) showing brown areas (yellow arrow) with peri- and inter-follicular scaling (blue arrow). B) Post-treatment polarized dermoscopic image (DermLite DL5, with 10× magnification) demonstrating a marked reduction in scaling.

**Figure 4:**
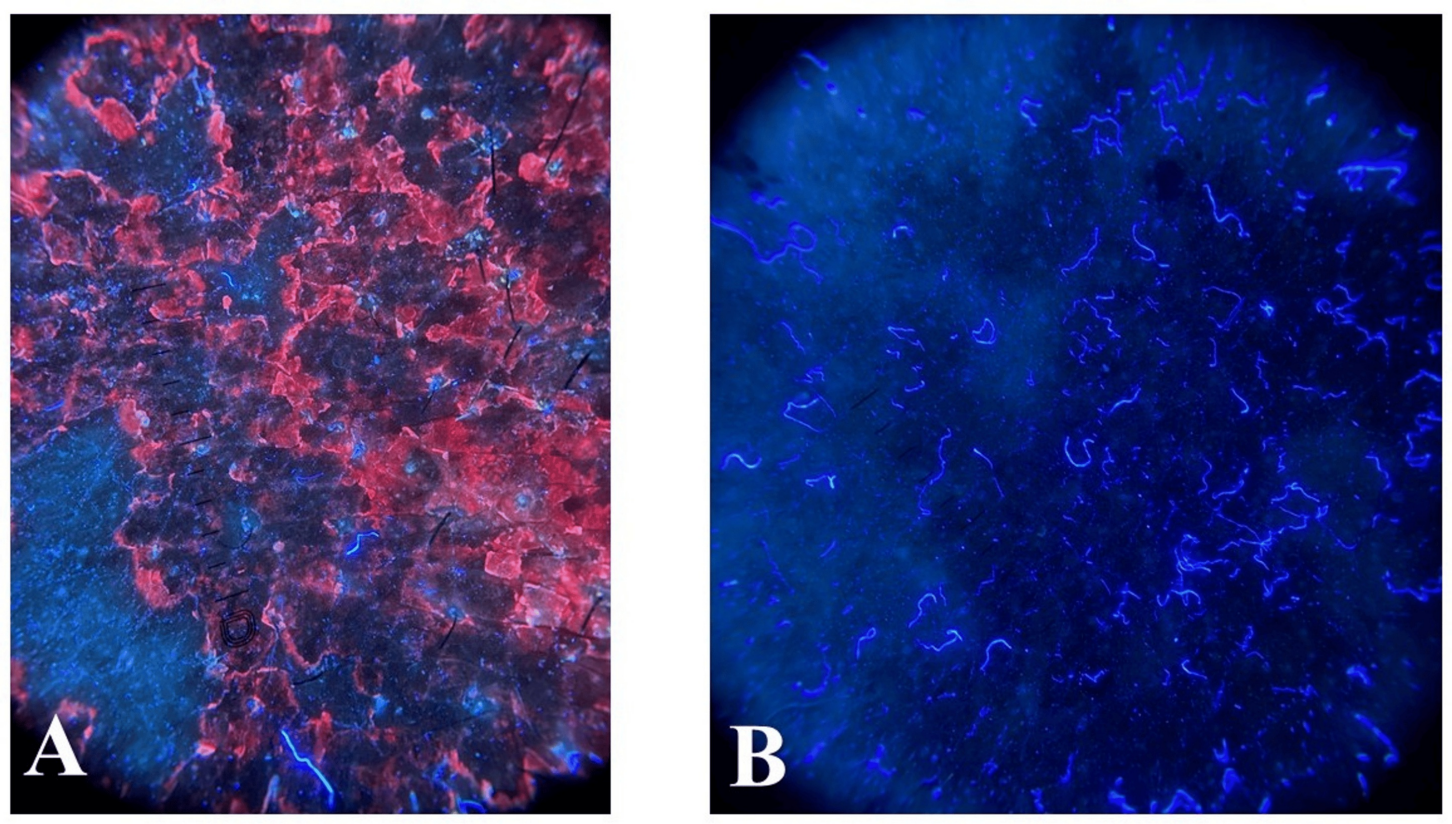
UV fluorescence (UVF) dermoscopy image A) Pre-treatment UV fluorescence (UVF) dermoscopic image (DermLite DL5, with 10× magnification and UV light) showing coral-red fluorescence characteristic of erythrasma. B) Post-treatment UV fluorescence (UVF) dermoscopic image (DermLite DL5, with 10× magnification and UV light) showing complete resolution of coral-red fluorescence following therapy.

Cutaneous examination showed well-circumscribed, brownish, scaly macules and patches, with mild erythema. The involved area was the axilla and groin (Figures 1A, 2A). There was no evidence of pustules, vesicles, or signs of secondary infection. Polarized dermoscopy of the lesion demonstrated brown areas with peri- and inter-follicular scaling (Figure 3A). UVF dermoscopy (DermLite DL5, with 10× magnification UV light) demonstrated characteristic coral-red fluorescence, localized to the affected skin in the axillae and groins (Figure 4A). A KOH preparation was negative for hyphae and spores, which made a diagnosis of fungal infection unlikely; in view of the clinical appearance, dermoscopic findings, and negative KOH, erythrasma was considered the most probable diagnosis. He was treated with combined topical and oral antibiotics: Whitolyn cream, mixed extemporaneously with fusidic acid, applied twice daily to the lesions, together with oral doxycycline 100 mg once daily for 20 days. Three weeks after commencing treatment, the lesions showed marked clinical improvement, with decreased pigmentation and scaling (Figures 1B, 2B). Polarized dermoscopy showed a reduction in scaling (Figure 3B). UVF dermoscopy showed complete disappearance of the coral-red fluorescence (Figure 4B), indicating both clinical and microbiological remission. The patient remained relapse-free at the three-month follow-up.

## Discussion

Erythrasma results from *C. minutissimum* infection, which produces an uncomplicated disease that is clinically challenging to recognize due to phenotypic overlap, because it resembles tinea versicolor and intertrigo in clinical presentation. Both entities involve intertriginous areas, exhibit similar reddish-brown pigmentation and scaling, and often lead to erroneous antifungal therapy [1,2,8]. The disease shows higher rates of occurrence in tropical regions that have warm and wet weather patterns, and particularly affects people who have diabetes, obesity, and compromised immune systems [9-11]. The diagnosis of this case proved difficult during standard examination, which shows how UVF dermoscopy serves as an essential diagnostic tool to establish more accurate diagnoses.

The coral-red fluorescence that appears under UV light results from bacterial production of coproporphyrin III, which functions as a distinctive porphyrin molecule in *C. minutissimum* [3,10]. The combination of UV light with high-resolution magnification in UVF dermoscopy enables clinicians to see the specific red fluorescence pattern of erythrasma, which appears as polygonal or structureless shapes [4,7]. The studies conducted by Pietkiewicz et al. showed that UVF dermoscopy enables clinicians to distinguish between erythrasma and tinea versicolor fungal infections [4,7,12]. Wood’s lamp examination functions as a basic diagnostic tool, but its restricted magnification, together with environmental light dependence, creates inconsistent results, which can result in diagnostic errors [4,10,13].

The bacterial origin of the infection became evident after the fluorescence signals disappeared when the patient received treatment with topical fusidic acid and oral doxycycline, and UVF dermoscopy demonstrated its value for tracking treatment response. Previous research studies showed similar results when antibacterial therapy achieved full clinical remission and fluorescence clearance for three months [1,5]. The combination of Whitolyn ointment (salicylic acid 3% + benzoic acid 6%) with oral doxycycline in erythrasma offers synergistic therapeutic action. Salicylic acid aids keratolysis and enhances antimicrobial penetration, while benzoic acid lowers skin pH, exerting bacteriostatic effects against *C. minutissimum*. Doxycycline inhibits bacterial protein synthesis via the 30S ribosomal subunit, providing systemic control. Together, they ensure comprehensive eradication of infection and reduce recurrence.

UVF dermoscopy distinctly highlights the coral-red fluorescence of *C. minutissimum*, alongside the bluish-green or yellow fluorescence of fungal elements, enabling clear delineation of mixed infections. This dual visualization enhances diagnostic precision and facilitates tailored antibacterial-antifungal therapy.

The research studies by Bhat et al. [6] and Sivakumar et al. [7] have shown that UVF dermoscopy continues to grow as a diagnostic tool for various infectious skin diseases, including erythrasma, pitted keratolysis, and trichomycosis. The diagnosis of erythrasma receives strong support from the agreement between fluorescence pattern, clinical morphology, and therapeutic response, even though this report does not include bacterial culture or histopathological evidence, which is a limitation. Future research incorporating microbiologic validation and fluorescence quantification may further substantiate the role of UVF dermoscopy in superficial bacterial infections.

## Conclusions

Erythrasma should be considered in the differential diagnosis of chronic, scaly dermatoses that fail to respond to antifungal therapy, as its clinical presentation often closely mimics superficial fungal infections. The application of UVF dermoscopy functions as an immediate, non-invasive diagnostic tool that provides both confirmation of diagnosis and monitoring capabilities for treatment evaluation. Integrating UVF dermoscopy into routine dermoscopic evaluation of intertriginous dermatoses could markedly improve diagnostic accuracy and reduce inappropriate antifungal prescriptions. The clinical implementation of UVF dermoscopy enables dermatologists to achieve better diagnostic results, which leads to decreased antifungal drug use and allows for timely and targeted treatment for erythrasma.
